# The Influence of Neurocognitive Impairment on HIV Risk Behaviors and Intervention Outcomes among High-Risk Substance Users: A Systematic Review

**DOI:** 10.3389/fpubh.2016.00016

**Published:** 2016-02-09

**Authors:** Roman Shrestha, Michael Copenhaver

**Affiliations:** ^1^Department of Community Medicine and Health Care, University of Connecticut Health Center, Farmington, CT, USA; ^2^Center for Health, Intervention, and Prevention, University of Connecticut, Storrs, CT, USA; ^3^Department of Allied Health Sciences, University of Connecticut, Storrs, CT, USA

**Keywords:** neurocognitive impairment, substance abuse, HIV risk behaviors, systematic review, HIV/AIDS, behavioral interventions

## Abstract

Neurocognitive impairment (NCI) among high-risk substance users poses a substantial barrier to reducing risk behaviors in this population. Previous work suggests that NCI is intertwined in a close, reciprocal relationship with risk behaviors. Not only does substance use worsen cognitive impairment but cognitive impairment may also reduce the efficacy of interventions aimed at reducing risk and improving medication adherence. In this systematic review, we examine the potential impact of substance abuse and cognitive functioning in the context of HIV risk behaviors and risk-reduction intervention outcomes. The findings thus far suggest that, in order to be effective, risk-reduction interventions must take into account the impact of NCI on learning, memory, and behavior.

## Introduction

Since the beginning of the HIV/AIDS epidemic, approximately 600,000 people have died of AIDS-related illnesses in the United States alone. Despite a wide array of preventive measures, the US is still facing a continuing epidemic, with approximately 50,000 new infections per year (1). Substance use – including alcohol, injection drug use (IDU), and non-IDU – remains an important risk factors fueling the epidemic. Although the number of new HIV infections attributed to IDU has declined significantly over time, rates continue to be high among specific sub-populations, including racial/ethnic minority groups (2). Beyond risk from injection-related behaviors (e.g., sharing needles), use of illicit drugs is an important driver of sexual transmission of HIV. Previous research has found that substance users engage in high-risk sexual behavior, including trading sex for drugs or money (3–5), having multiple sex partners (6–8), and unprotected intercourse (7–9). Therefore, there is an urgent need for interventions targeting substance use-related behavioral changes in order to reduce rate of HIV incidence in this population.

Recent studies exploring the effects of substance use on the brain have demonstrated a variety of adverse effects on the central nervous system (CNS) resulting in neurocognitive impairment (NCI) symptoms (10–14). Indeed, studies have shown an extensive overlap between the brain regions and neural processes involved in substance addiction and those that are known to be of relevance for cognitive functions like learning, memory, and reasoning. The repeated exposure to abusive drugs produces significant structural and functional abnormalities in the brain, predominantly in the frontal cortex and temporal lobe, areas considered crucial for executive control (15–21). This cognitive shift, followed by continued substance abuse, may play a role in the development of addiction and exacerbate the difficulty of sustaining abstinence.

Substance abuse and the presence of neurocognitive deficits are independently associated with increased drug- and sex-related risk behaviors and HIV intervention outcomes in studies that have examined high-risk HIV substance using samples (3–9, 22–25). Some research has suggested synergistic deleterious impacts of substance use and NCI on HIV-related risk behaviors (24, 26). Importantly, the impairment in cognitive functioning influenced by continued substance use among substance abusers may also prevent normal acquisition and retention of intervention content delivered through behavioral risk-reduction intervention approaches (22, 27, 28). Thus, understanding the relationship between substance abuse, NCI, and HIV risk factors is crucial to successfully intervening against a range of risk behaviors among substance abusers. This systematic review is aimed at exploring the relevant research literature to more clearly examine the possible impact of NCI on HIV risk as well as HIV intervention efforts targeting high-risk HIV substance users.

## Methods

### Search Methods for Identification of Studies

The literature review was restricted to peer-reviewed original human research articles and dissertations that focused on the effects of cognitive functioning on HIV risk behaviors. Searches included relevant English language papers published in 1981–2014. Relevant studies were located using several search strategies. First, PubMed, ProQuest Dissertations, and Theses were searched using Boolean operators:: {[(HIV risk behaviors) OR (sexual risk behaviors) OR (substance abuse risk behavior) OR (drug abuse risk behavior) OR (needle shar*) OR (condom use) OR (HIV knowledge)] AND [(neuropsychol*) OR (cognitive deficit) OR (neurocognitive disorder) OR (neurocog*) OR (executive function) OR (attention deficit) OR (memory deficit) OR (HIV-associated neurocognitive disorders) OR (visual motor function) OR (spatial problem solving) OR (psychomotor function) OR (speed of processing) OR (visual attention) OR (visual learning) OR (working memory deficit) OR (social cognition)] AND [(substance use*) OR (drug use*) OR (drug addict*) OR (drug dependent) OR (IDU) OR (injection drug use*) OR (illegal drug use*) OR (opiate user) OR (heroine user) OR (street drug user)]}. Both a combined text word and Medical Subject Headings (MeSH) terms were used to identify relevant papers. Second, a secondary search was conducted that involved checking the reference sections of relevant review papers and google scholar for articles that may have been missed in the initial computerized search.

### Study Selection and Inclusion/Exclusion Criteria

In conducting this systematic review, peer-reviewed studies were included if they met all of the following criteria: (a) participants were high-risk substance users; (b) assessed participants’ NCI status; (c) assessed substance- or sexual-related HIV risk behavior outcome(s); and (d) were published in English. The search was not limited to any particular geographic area or region, and there were no restrictions imposed on the age of subject populations. Studies were excluded if they focused on sexual and drug use transmission risk but not on cognitive impairment. We also excluded pharmaceutical studies.

**Figure 1:**
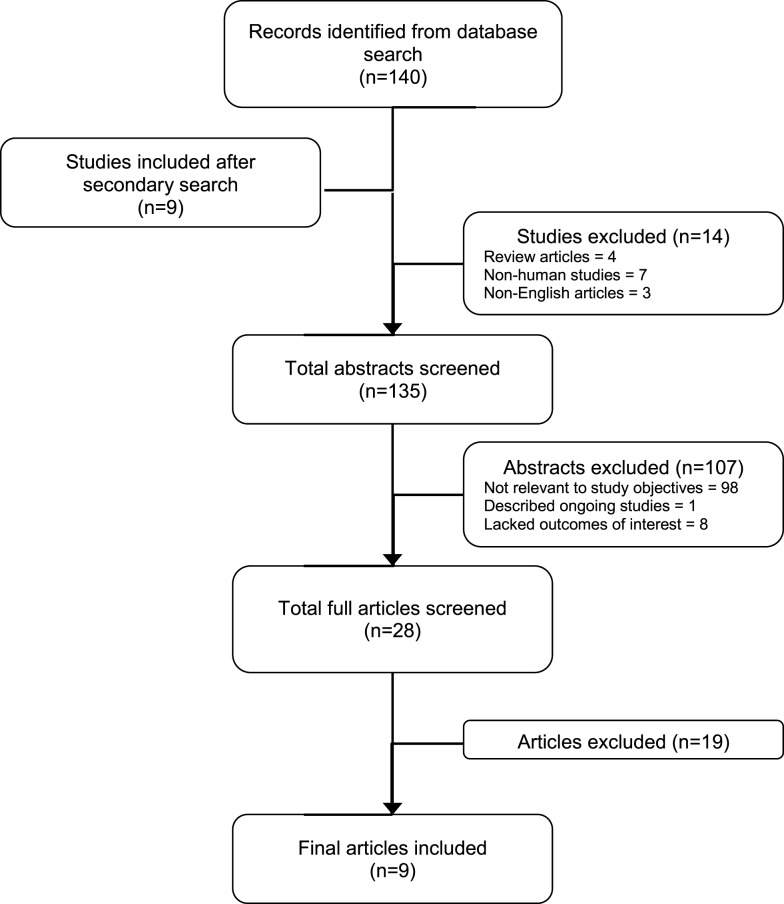
**Summary of study selection process**.

A total of 140 articles were retrieved as of September, 2015. After nine additional articles were found in the reference section of the relevant journal articles, we had 148 articles for preliminary review. Of these, 14 were subsequently excluded because they were either review articles (4), were not human studies (7), and non-English articles (3), leaving 135 records for further review (Figure 1). After inspecting study titles and abstracts, we found that 107 records were not directly relevant to the study objectives (98), described ongoing studies with no data published (1) or lacked the stated outcomes of interest (8), leaving 28 records for further, more detailed, review. A full-text copy of this subset of studies was obtained and assessed for inclusion. Nineteen full-text records were excluded because they did not investigate the variables of interest. Thus, a total of nine studies were included for this review (Figure 1).

### Data Extraction and Management

Data concerning study characteristics, participant characteristics, study design characteristics, and outcome characteristics were independently abstracted from relevant studies by two trained reviewers (Roman Shrestha and Michael Copenhaver). Any discrepancies were resolved by discussion.

A standardized form was used for data extraction. *Article characteristics* included such dimensions as (a) authors, (b) year of publication, (c) year of data collection, (d) sample size, (e) location of study, and (f) whether a theoretical framework guided the interventional design, if applicable. *Participant characteristics* included (a) age, (b) race/ethnicity, (c) gender distribution of participants, and (d) sample type. *Study design characteristics* included (a) type of study design (e.g., randomized controlled trial), (b) type of control group, and (c) type of neurocognitive deficit measure. *Outcome characteristics* included influence of NCI on HIV risk behaviors, including (a) knowledge about HIV, (b) motivation to change behaviors, (c) self-reported condom use (male or female condoms), (d) self-reported number of sexual partners, (e) self-reported IDU pattern, and (f) self-efficacy (assessing self-confidence related to condom application skills, sexual practices, and needle cleaning skills after intervention).

## Results

### Description of Studies

A total of nine studies met the review inclusion criteria (Table 1). Studies were conducted between 1999 and 2014, with the majority (77.8%) taking place within the past decade (2004–2014). In terms of methods, over one-third of the studies (33.3%) were cross-­sectional, 33.3% longitudinal, 22.2% were randomized controlled trial, and 11.1% were case–control. Studies collected data through face-to-face interviews (66.6%) or self-administered surveys (33.3%). Seventy-seven percent (*k* = 7) of the studies mentioned the specific measure used to assess neurocognitive deficit of the participants, whereas 22.2% (*k* = 2) did not. HIV-related knowledge among samples was assessed in two studies (22.2%), motivation to change risk behaviors in two studies (22.2%) and self-efficacy in two studies (22.2%). Sexual risk was measured in terms of unprotected sex in seven studies (77.8%), condom use in seven studies (77.8%), and having multiple sexual partners in two studies (22.2%).

**Table 1:** **Summary of studies included in the systematic review**.

Study	Study location	Study site	Sample size (*N*)	Gender distribution		Study population	Study design	Summary of findings
				Female	Male			
Albein-Urios et al. (29)	Granada, Spain	Substance Abuse Treatment Clinic	72	NR	NR	Cocaine dependent vs. pathological gamblers	Case–control	Peak amount of cocaine use was negatively correlated with working memory and response inhibition performance.
Black et al. (30)	Connecticut, USA	Community Mental Health Center	51	31	20	Substance dependent	Cross-sectional	Rash-spontaneous impulsivity was not associated with risky sexual behaviors
Dolezal et al. (31)	New York, USA	HIV Center for Clinical and Behavioral Studies	144	0	144	IDU	Longitudinal	Presence of HIV-related symptoms and neuropsychological impairment were associated with lower sexual risk. Neurological impairment and depression were not associated with sexual risk behavior
Gonzalez et al. (23)	Chicago, USA	Chicago Metropolitan Area	263	NR	NR	HIV+ and HIV-substance dependent individuals	Cross-sectional	Continued risk behavior among HIV+ drug users may be driven by sensation seeking (a personality trait common among drug users); however, the impact of executive functions is less clear
Mitchell et al. (32)	Maryland, USA	Baltimore Site of the International Neurobehavioral HIV Study	229	78	151	IDU	Longitudinal	African American IDUs, especially those with lower cognitive functioning, compared with White IDUs, were less likely to share drugs and other injection equipment; Cognitive performance moderated the effect of knowing someone who had died from AIDS on engaging in risky injection drug use behaviors, such that individuals who had lower cognitive scores and knew someone who had died from AIDS were more likely to be in the high-risk group for injection behavior
Sadeghi-Najafabadi (27)	Connecticut, USA	Methadone Clinic	280	151	129	HIV-individuals opioid dependent drug users receiving MMT	RCT	Participants with lower levels of NCI who specifically receive the CHRP intervention demonstrate more HIV risk reduction skill development while, less improvement from the intervention content was seen among the intervention group participants with higher levels of NCI
Schuster et al. (25)	Chicago, USA	Chicago Metropolitan Area	66	25	41	Cannabis users	Cross-sectional	Contrary to hypotheses, worse episodic memory also significantly predicted higher overall sexual-risk and decreased safe-sex practices
Stacy et al. (33)	California, USA	Junior High School	579	NR	NR	Men and women in Los Angeles County	Longitudinal	Memory association predicted unprotected sex in the high-risk but not the low-risk sample The implicit cognition measure used in the present study predicted unprotected sex in this sample even when other potentially strong predictors were controlled for in the analysis (e.g., drug use, sensation seeking)
Worley et al. (28)	California, USA	VA Substance Abuse Mental Illness (SAMI) Program	197	19	178	Veterans receiving treatment for AODD and MDD	RCT	More severe baseline NCI predicted poorer alcohol and drug use outcomes via lower self-efficacy, lower 12-step affiliation, & greater depressive symptoms

*NR, not reported*.

Overall, 1,836 substance users were enrolled across the study. Study sample sizes ranged from 51 to 579, with the average number of 204 per study. The average age across samples was 36.8 years (range = 15.8–49.3; *k* = 9). Study participants were all HIV-negative substance users randomly recruited from venues that served active substance users, including substance abuse treatment clinics, service organizations, or defined communities. Less than half (44.4%) of the studies provided monetary incentives for study participation. A complete summary of the outcome measures generated from each citation appears in Table 1. The results by outcome were as follows.

### Neurocognitive Impairment and Its Influence on HIV Risk Behaviors

The majority of the studies reviewed in the present study found a significant association between a higher level of NCI and a greater level of HIV-related risk behaviors among substance users. A recent study by Worley et al. on the effects of NCI on substance uses among adults in treatment for alcohol or drug dependence found that more severe NCI predicted poorer alcohol and drug use outcomes via lower self-efficacy (i.e., confidence to resist use of alcohol or drugs across a variety of high-risk situations) (28). Interestingly, among a sample of heroin IDUs who were HIV-seronegative, aged between 18 and 30 years, Mitchell et al. found that cognitive performance had a moderating effect on IDU behaviors, such as individuals who had lower cognitive scores and knew someone who had died from AIDS were more likely to report higher-risk injection behavior (i.e., shared needles or used the same cooker, cotton, or rinse water) (32).

In a longitudinal study, Stacy et al. showed that implicit cognition indicator, which refers to the learning, memory, and performance processes, which take place without the subject’s conscious awareness, was an independent predictor of lack of condom use in a high-risk sample (33). Implicit cognition predicted unprotected sex in this sample even when other potentially strong predictors were controlled (e.g., drug use, sensation ­seeking) (33). Likewise, Schuster et al. found both direct and indirect associations between aspects of inhibitory control and risky sexual behaviors (RSBs) (25). The same study also found that the amount of recent cannabis use was associated with overall sexual-risk, as expected. The relationship between amount of recent cannabis use and overall RSBs, however, was moderated by performance on decision-making, with significant relationships emerging only among those who performed more poorly. This supports the notion that neurocognitive functioning, particularly in the domains of risk-taking and episodic memory, may influence the degree to which cannabis users engage in RSBs (25).

More recently, Albein-Urios et al. compared the cognitive performance of cocaine-dependent individuals (CDI) with that of pathological gamblers (PG) (29). The study found that CDI, as compared to PG, had elevated scores on Negative Urgency and poorer performance on working memory. Correlational analyses showed significant negative associations between performance on the working memory and response inhibition tasks and peak amount of cocaine use (29). The finding of this study that cocaine dependence is specifically associated with working memory deficits is in agreement with our hypothesis.

Although the vast majority of studies reported some degree of influence of NCI on HIV-related risk behaviors, we also identified studies that suggested otherwise. For example, a cross-sectional study among poly-drug users from the Chicago metropolitan area, seeking services from various community clinics, did not find any significant relationships between neurocognitive performance and self-reported risky sexual practices for the overall subject sample (23). Also, Black et al. reported similar findings among psychiatric outpatients with a comorbid substance abuse dependence disorder in a community mental health center in Connecticut, USA. They reported that cognitive impulsivity was not found to have a significant correlation with any RSBs (30).

Furthermore, similar finding has been found among a sample of IDUs in New York City who are infected with HIV. In a longitudinal study, Dolezal et al. found no significant association between neurological assessment outcomes and any of the risk behaviors, including less unprotected sex, abstinence, and lower risk index scores. Lower scores on the Selective Reminding Test, however, were associated with abstinence and lower risk index scores (31).

### Neurocognitive Impairment and Its Influence on Intervention Outcomes

Researchers recently explored the association between NCI and risk reduction intervention outcomes among chronic substance users participating in methadone-maintained drug treatment (27). Following the intervention, improvements in treatment outcomes were negatively associated with NCI among the study participants. This finding showed that participants with higher levels of neurocognitive deficits demonstrated lesser HIV risk reduction skills development compared with participants with lower levels of NCI. The same study also found that higher degree of NCI was associated with lower self-efficacy about sex-risk reduction behavior (i.e., purchasing condoms) as well as more difficulty learning HIV risk reduction skills (i.e., needle cleaning skills) (27).

## Discussion

We reviewed studies that included data on the influence of NCI on HIV risk behaviors and intervention outcomes among high-risk substance users. Consistent with our hypotheses, we found both direct and indirect associations between NCI and risk behaviors and intervention outcomes (25, 27–29, 32, 33). The nature of findings linked to neurocognitive deficits suggests that this may negatively influence risk-taking behavior. For example, impaired executive function impedes rational decision-makingwhich may prevent individuals from making safe sexual choices. Similarly, slowed information processing function may prevent the timely, appropriate consideration of risk variables during decision-making instances (34).

Neurocognitive impairment may also play a substantial role in how drug dependent individuals respond to HIV interventions (27, 28). Traditional “behavioral” HIV prevention strategies tend to place a high demand on numerous cognitive abilities such as attention, learning, memory, and information processing (35). As such, neurocognitive deficits may function as a confounding variable with regard to a range of intervention outcomes. Moderate levels of impairment that is common among substance users may impede the presumed attainment and retention of behavioral content provided in traditional intervention and treatment programs (34).

Our results support the notion that cognitive functioning, particularly in the domains of executive function, decision-making, working memory, and cognitive impulsivity, may influence the degree to which substance users engage in risky behaviors (3, 7, 23, 25, 27). Importantly, these findings persisted even after accounting for confounding variables (e.g., substance use), suggesting that the relationships between neurocognitive functioning and risk behaviors are not simply due to severity of substance use. Some results, however, also indicate that the presence of NCI may not confer increased risk behavior universally. Instead, the contribution of cognitive deficits on risk behavior may vary across ethnicities and/or geographic location, socioeconomic status, gender, and sexual orientation, thus precluding universal generalizations, as might be expected (23, 30, 31).

Some of the limitations of this systematic review should be acknowledged. First, the review of articles was restricted to peer-reviewed journal articles published in English. This may have likely biased our selection of articles toward predominantly English-speaking countries. In addition, we initially screened only abstracts to determine whether the study investigated the impact of cognitive deficits on HIV risk behavior outcomes. Thus, any secondary outcomes and analyses pertinent to our areas of interest not mentioned within the abstracts may have been excluded from this review. Many risk behaviors were self-reported by the study participants, often at much later time points, making their response open to bias and social desirability factors. In addition, due to a reliance on primarily cross-sectional study designs, it is not possible to drawn causal inferences regarding the independent influence of NCI.

## Conclusion and Future Directions

The findings of this study indicate that the majority of the available research has shown a close and reciprocal association between NCI and increased HIV risk behaviors among high-risk substance users. The literature also suggested that NCI is associated with how well these same risk individuals respond to interventions aimed at reducing risk behaviors. The findings from this review suggest that more attention needs to be given to screening for NCI among this population, and appropriately accommodating them in the delivery of HIV risk-reduction interventions. Though untested to date, previous reviews have suggested that behavioral HIV prevention strategies integrating cognitive remediation strategies, multi-modal presentation of information, and a focus on real-world applications may be useful when incorporated into behavioral risk reduction strategies among individuals with cognitive deficits (34). As such, future studies should examine the feasibility and direct impact of incorporating specific behavioral intervention strategies designed to accommodate the moderate levels of NCI – thus allowing greater potential benefit – among high-risk substance users.

## Author Contributions

Conceived and designed the study: RS and MC. Analyzed the data: RS and MC. Wrote the paper: RS. Proof read the paper: RS and MC. Final approval: RS and MC.

## Conflict of Interest Statement

The authors declare that the research was conducted in the absence of any commercial or financial relationships that could be construed as a potential conflict of interest.
